# Two Pediatric Cases of Primary Ciliary Dyskinesia Caused by Loss-of-Function Variants in Oral-Facial-Digital Syndrome Gene, *OFD1*

**DOI:** 10.1155/2024/1595717

**Published:** 2024-08-09

**Authors:** Yifei Xu, Yuki Tsurinaga, Tsubasa Matsumoto, Ryuji Muta, Taichi Yano, Hiroshi Sakaida, Sawako Masuda, Koki Ueda, Guofei Feng, Shimpei Gotoh, Satoru Ogawa, Makoto Ikejiri, Kaname Nakatani, Mizuho Nagao, Masaki Tanabe, Kazuhiko Takeuchi

**Affiliations:** ^1^ Department of Otorhinolaryngology-Head and Neck Surgery Mie University Graduate School of Medicine, Tsu, Japan; ^2^ Department of Pediatrics Osaka Habikino Medical Center, Osaka, Japan; ^3^ Department of Pediatric Infection and Immunology Fukuoka Children's Hospital, Fukuoka, Japan; ^4^ Department of Allergy and Respiratory Medicine Fukuoka Children's Hospital, Fukuoka, Japan; ^5^ Faculty of Medicine Mie University, Tsu, Japan; ^6^ Department of Otorhinolaryngology National Hospital Organization Mie National Hospital, Tsu, Japan; ^7^ Center for iPS Cell Research and Application Kyoto University, Kyoto, Japan; ^8^ Electron Microscopy Research Center Mie University Graduate School of Medicine, Tsu, Japan; ^9^ Department of Clinical Laboratory Mie University Hospital, Tsu, Japan; ^10^ Department of Medicine Iga City General Hospital, Iga, Japan; ^11^ Institute for Clinical Research National Hospital Organization Mie National Hospital, Tsu, Japan

## Abstract

Primary ciliary dyskinesia (PCD) is a hereditary disease caused by genes related to motile cilia. We report two male pediatric cases of PCD caused by hemizygous pathogenic variants in the OFD1 centriole and centriolar satellite protein (*OFD1*) gene. The variants were NM_003611.3: c.[2789_2793delTAAAA] (p.[Ile930LysfsTer8]) in Case 1 and c.[2632_2635delGAAG] (p.[Glu878LysfsTer9]) in Case 2. Both cases had characteristic recurrent respiratory infections. Neither case had symptoms of oral-facial-digital syndrome type I. We identified a variant (c.2632_2635delGAAG) that has not been previously reported in any case of *OFD1*-PCD.

## 1. Introduction

Primary ciliary dyskinesia (PCD) is a hereditary disorder caused by pathogenic variants of genes related to motile cilia. PCD is characterized by persistent productive cough and is associated with various symptoms including recurrent infections of the upper and lower respiratory tract due to inefficient respiratory mucociliary clearance caused by ciliary beating disorders. Other clinical manifestations include otitis media with effusion, infertility, and left-right laterality defects [1].

More than 50 PCD causative genes have been reported and most cases are inherited in an autosomal recessive manner, while X-linked inheritance is rare. OFD1 centriole and centriolar satellite protein (*OFD1*), a gene on the X chromosome, is associated with oral-facial-digital syndrome type I (OFDSI), which is an X-linked dysmorphology syndrome related to both primary and motile cilia, and is one of the causative genes for X-linked PCD [2].

*OFD1* has long been known as the causative gene of primary ciliopathy in OFDSI with a prevalence of 1/50,000 live births [3], and OFDSI is characterized by dysmorphology of the face, oral cavity, and digits with a high degree of phenotypic variability [4]. Involvement of the central nervous system is frequent, including central nervous system malformations, mental retardation, and selective cognitive impairment [4]. Renal cysts are also common in OFDSI patients, with an overall prevalence of 43%. Renal impairment can be present at birth; however, its prevalence increases with age and most cases develop cystic kidney disease in adulthood [4, 5].

In 2006, Budny et al. [6] reported severely uncoordinated and disorganized cilia movement that was observed by high-speed video microscopy of patients' nasal epithelium, which firstly supported the role of *OFD1* in respiratory motile ciliary function [6]. Patients with *OFD1* variants were reported to have features of PCD. Hannah et al. described the first three PCD cases with pathogenic variants in *OFD1* [7].

Here, we report two male Japanese pediatric *OFD1*-PCD cases.

## 2. Case Report

### 2.1. Case 1

We received a consultation regarding a boy aged 1 year and 7 months with recurrent pneumonia, and blood samples from the case as well as his parents were sent to our department for genetic testing. He was born at 39 weeks. He had nasal obstruction and rhinorrhea immediately after birth. He was not admitted to a neonatal intensive care unit. He had productive cough during the neonatal period and rhinitis that persisted for more than 1 year. He was hospitalized four times due to pneumonia at 2 months, 4 months, 13 months, and 16 months of age, respectively. He did not have *situs inversus* or a congenital heart defect. He did not have facial dysmorphology or polysyndactyly. His Primary CiliAry DyskinesiA Rule (PICADAR) score [8] was 5 (full-term birth, neonatal respiratory symptoms, and rhinitis), indicating an 11% probability of PCD.

**Figure 1:**
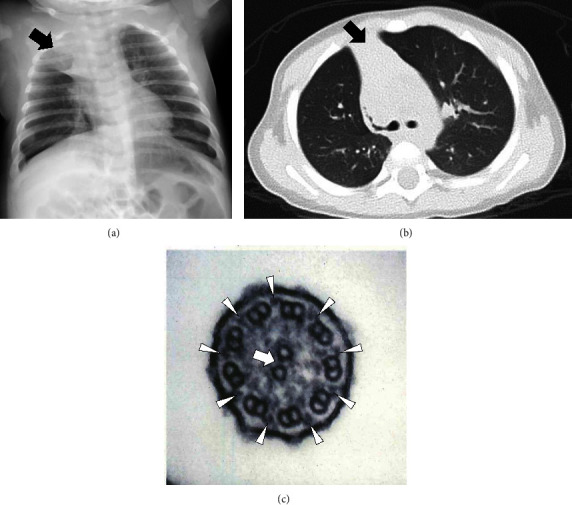
Imaging findings in Case 1. (a) Chest X-ray at 2 months of age showing *situs solitus* and consolidation (black arrow) with a loss of lung volume in the right upper lobe. (b) Chest computed tomography showing consolidation (black arrow) with an air bronchogram in the right upper lobe. (c) Transmission electron microscopy findings of a biopsy specimen from the nasal mucosa of case 1 showing normal ciliary axoneme ultrastructure. The cilium consists of one pair of central microtubules (white arrow) surrounded by nine pairs of well-arranged peripheral microtubules. The outer dynein arms (arrowheads) are presented.

Chest X-ray at 2 months old showed *situs solitus* and consolidation in the right upper lobe (Figure 1(a)). Chest computed tomography at 3 months of age showed consolidation with an air bronchogram in the right upper lobe (Figure 1(b)).

**Figure 2:**
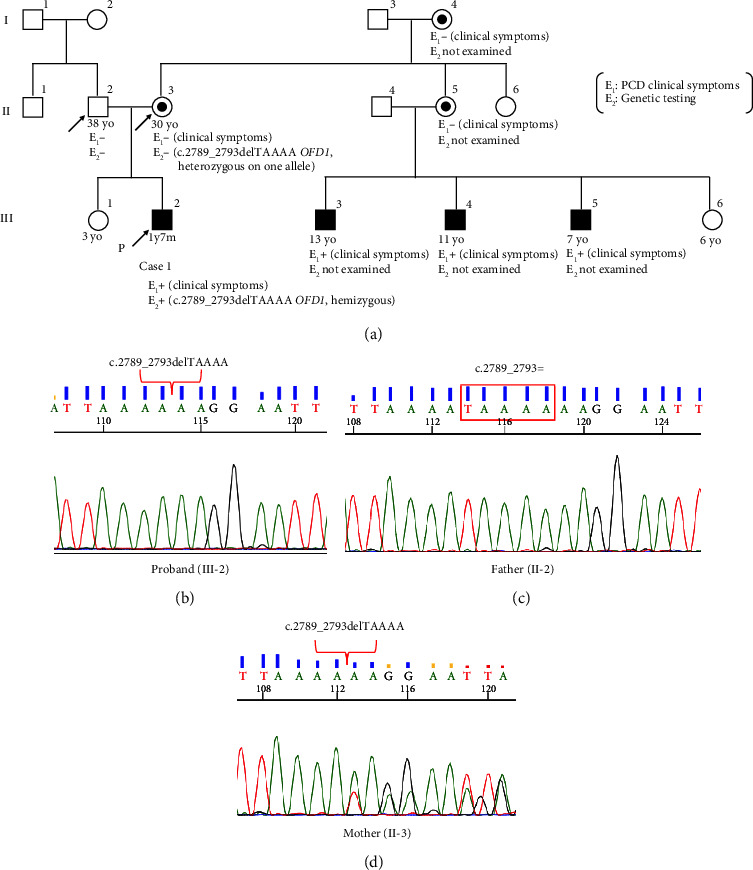
Family pedigree of case 1 with the results of Sanger sequencing. (a) Family pedigree of case 1. III-2 is the proband (Case 1) and his mother (II-3) is a carrier. III-3, -4, and -5 had symptoms similar to those of III-2, but genetic analysis has not been performed on these individuals. I-4 and II-5 are presumed to be carriers. (b) The proband (III-2) has a hemizygous variant in *OFD1*. (c) His father (II-2) does not carry the same variant. (d) The proband's mother (II-3) is a carrier of the same heterozygous deletion.

Transmission electron microscopy of respiratory cilia from the nasal mucosa showed normal ciliary axoneme ultrastructure (Figure 1(c)). His parents and 3-year-old sister did not have PCD or OFDSI. However, three sons of his maternal aunt all had similar clinical symptoms as the proband (they have not undergone genetic testing) (Figure 2(a)). Whole-exome sequencing identified a frameshift deletion in *OFD1* (NM_003611.3: c.[2789_2793delTAAAA], p.[Ile930LysfsTer8]) in the proband. Sanger sequencing confirmed that his father did not carry this variant (Figure 2(c)), while this heterozygous deletion in *OFD1* was found in the proband and his mother (Figures 2(b) and 2(d)).

### 2.2. Case 2

A 5-year-old boy was referred to our department because of persistent wet cough from 1 month of age. He was born at 38 weeks. He was not admitted to a neonatal intensive care unit. He had cough, sputum, and wheezing since 1 month of age. Poor weight gain was noted at 4 months of age. He was hospitalized due to asthma and pneumonia at the age of 3 months and 30 days, and *situs inversus* was noted at that time. He was hospitalized again at the age of 5 months due to bronchitis. He had secretory otitis media and chronic sinusitis. He did not have facial dysmorphology or polysyndactyly. His PICADAR score was 6 (*situs inversus*, rhinitis, and otitis media), indicating a 24% probability of PCD. Nasal nitride oxide production was 31.5 nL/min.

**Figure 3:**
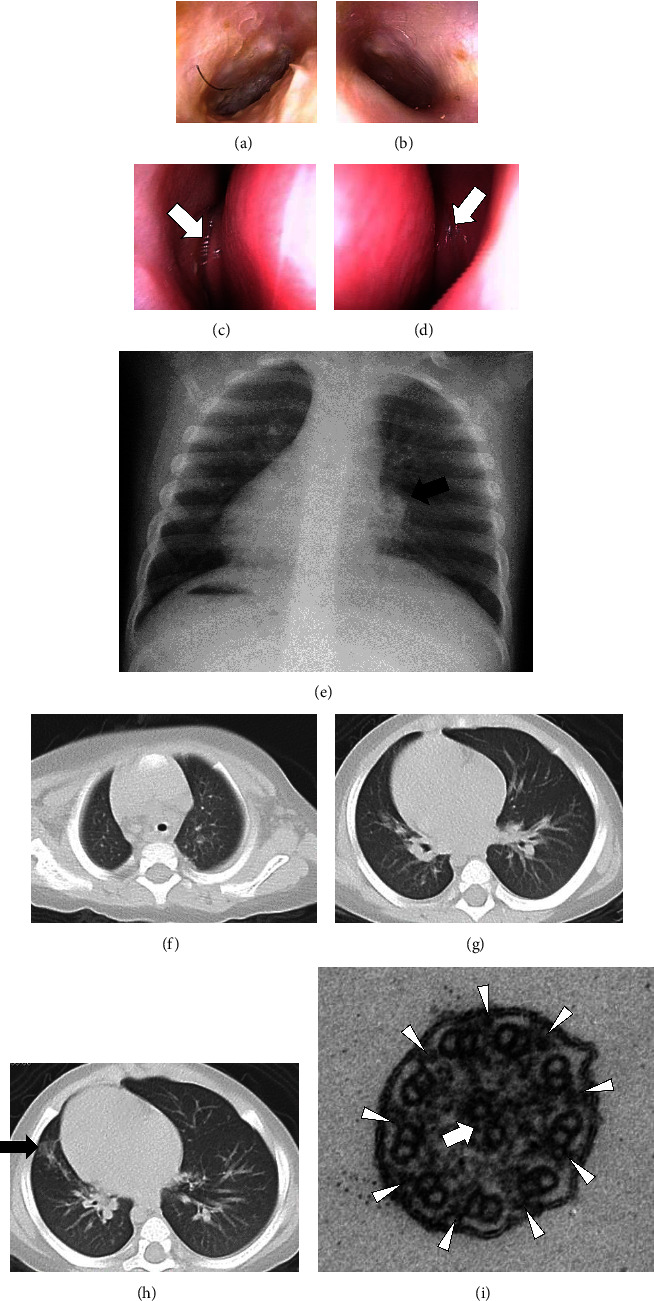
Imaging findings in Case 2. Ear endoscopy showing thickened bilateral tympanic membranes without a cone of light in the (a) right and (b) left ears. The middle nasal meatus is occluded with mucus secretion (white arrows) in the (c) right and (d) left nasal cavities. (e) Chest X-ray at 4 months of age showing *situs inversus totalis* and increased bronchovascular shadows (black arrow). Chest computed tomography of the (f) upper, (g) middle, and (h) lower lobes showing consolidation with ground glass opacity (black arrow) in the right lower lobe. (i) Transmission electron microscopy findings of Case 2. The cilium consists of one pair of central microtubules (white arrow) surrounded by nine pairs of well-arranged peripheral microtubules. The outer dynein arms (arrowheads) are presented.

The bilateral tympanic membranes were thickened and the cone of light was absent (Figures 3(a) and 3(b)). The middle nasal meatus was occluded bilaterally on nasal endoscopy (Figures 3(c) and 3(d)). Chest X-ray at 4 months of age showed *situs inversus* and increased bronchovascular shadows (Figure 3(e)). Chest computed tomography at 1 year of age showed consolidation with some ground glass opacity in the right lower lobe (Figures 3(f), 3(g), and 3(h)).

**Figure 4:**
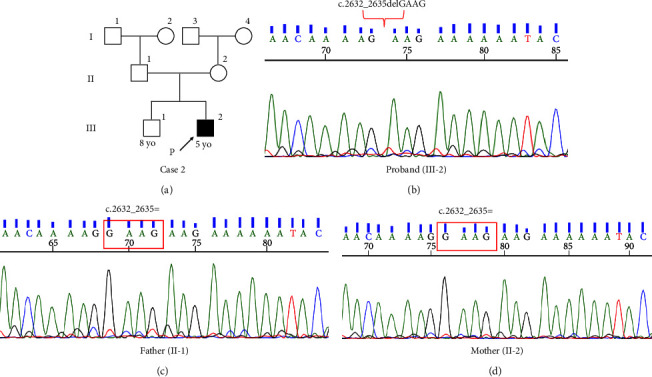
Family pedigree of Case 2 with the results of Sanger sequencing. (a) Family pedigree of Case 2. III-2 is the proband. The parents are not carriers. (b) The proband (III-2) has a *de novo* deletion variant in *OFD1*. (c) Neither his father (II-1) nor (d) mother (II-2) carries the same variant.

Transmission electron microscopy of respiratory cilia from the nasal mucosa showed the normal ciliary axoneme ultrastructure (Figure 3(i)). His parents and elder brother did not present with any symptoms of PCD or OFDSI (Figure 4(a)). Whole-exome sequencing identified a frameshift deletion in *OFD1* (NM_003611.3: c.[2632_2635delGAAG], p.[Glu878LysfsTer9]). Sanger sequencing confirmed that only the proband carried the variant (Figure 4(b)), while neither of his parents carried the same variant (Figures 4(c) and 4(d)).

## 3. Discussion

We report two male Japanese pediatric PCD cases without associated OFDSI symptoms caused by *OFD1*. The variant of Case 1 was the same as that of an *OFD1*-PCD case reported by Hannah et al. in 2019 [7]. The variant in Case 2 has not been previously reported in any other *OFD1*-PCD case. The allele frequency of both variants is unknown.

The frameshift variant identified in Case 1 was localized in exon 21 of *OFD1* while that of Case 2 was in exon 20. Consistent with these observations, Bukowy-Bieryllo et al. [9] reported four *OFD1*-PCD patients with truncating variants localized in exons 20 and 21 who all lacked OFDSI symptoms. The different locations of *OFD1* variants cause three different syndromes: OFDSI (exons 2–17), Simpson–Golabi–Behmel syndrome type 2 (exon 16), and Joubert syndrome 10 (exons 17–22). The severity of the clinical phenotype generally decreases as variants are localized closer to the C-terminus [9]. The C-terminal part of *OFD1*, which includes exons 16–23 (amino acids 719–991), contains five intrinsically disordered regions that are involved in protein-protein interactions. The C-terminal part of OFD1 interacts with protein partners and is important for the biogenesis of motile cilia. Changes in the intrinsically disordered regions can affect the binding of OFD1 with its protein partners and consequently lead to motile cilia dysfunction. Bukowy-Bieryllo et al. [9] confirmed that variants in exon 21 do not cause nonsense-mediated decay but may only lead to truncation of the C-terminal part. This may explain why the current two cases did not have OFDSI symptoms but presented with PCD symptoms.

Hannah et al. [7] reported three *OFD1*-PCD cases, and one of the patients carried the same frameshift variant as the present Case 1. Case 1 had inherited this variant maternally while that in the case reported by Hannah et al. was a *de novo* variant [7]. Heterozygous mutations of *OFD1* are often *de novo* and approximately 75% of affected females have a *de novo* variant [5, 10]. Our Case 2 also had a *de novo* variant.

OFD1 protein localizes to the centrosome and basal body of primary cilia [11]. An absence of cilia at the embryonic node and laterality defects are observed in male *Ofd1* knockout mice [12]. *Ofd1*-defective zebrafish show a bent body, laterality defects, and edema [13]. These findings indicate that *Ofd1* is required for primary cilia ciliogenesis and left-right specification. Consistent with the literature, our Case 2 also showed *situs inversus*.

At the centrosomal level, OFD1 is a negative regulator of centriole elongation [14] and interacts with other OFD proteins to initiate ciliogenesis and control centriole length. [15] Knockdown of *OFD1* induces hyperelongated centrioles and affects membrane anchoring, leading to the absence of cilia or abnormal cilium length. [16] Bukowy-Bieryllo et al. [9] found that *OFD1*-PCD patients have longer respiratory cilia than other PCD patients by high-speed video analysis and immunostaining. However, *OFD1*-PCD patients have normal ciliary axoneme architecture according to axonemal marker staining. Consistent with the study of Bukowy-Bieryllo et al. [9] the two cases reported here showed normal respiratory ciliary axoneme ultrastructure (Figures 1(c) and 3(i)).

## 4. Conclusion

We report two male Japanese pediatric PCD cases without associated OFDSI symptoms caused by frameshift variants in exons 20 and 21 of *OFD1*. In addition, we identified a variant (c.2632_2635delGAAG) that has not been reported in any *OFD1*-PCD case.

## Data Availability

The datasets generated and analysed during the current study are available from the corresponding author upon reasonable request.
